# The durability of minimally invasive lumbar decompression procedure in patients with symptomatic lumbar spinal stenosis: Long‐term follow‐up

**DOI:** 10.1111/papr.13020

**Published:** 2021-05-13

**Authors:** Nagy Mekhail, Shrif Costandi, George Nageeb, Catherine Ekladios, Ogena Saied

**Affiliations:** ^1^ Evidence‐Based Pain Management Research Cleveland Clinic Cleveland OH USA

**Keywords:** back pain, *mild*®, minimally invasive lumbar decompression, pain relief, spinal stenosis, surgical lumbar decompression

## Abstract

**Background:**

Minimally invasive lumbar decompression (*mild* ^®^) has been shown to be safe and effective for the treatment of lumbar spinal stenosis patients with hypertrophic ligamentum flavum as a contributing factor. This study examines the long‐term durability of the *mild* procedure through 5‐year follow‐up. Pain relief and opioid medications utilization during 12‐month follow‐up were also assessed.

**Methods:**

All patients diagnosed with lumbar spinal stenosis secondary to ligamentum flavum hypertrophy who underwent *mild* from 2010 through 2015 at the Cleveland Clinic Department of Pain Management were included in this retrospective longitudinal observational cohort study. The primary outcome measure was the incidence of open lumbar decompression surgery at the same level(s) as the *mild* intervention during 5‐year follow‐up. Secondary outcome measures were the change in pain levels using the Numeric Rating Scale and opioid medications utilization using Morphine Milligram Equivalent dose per day from baseline to 3, 6, and 12 months post‐*mild* procedure. Postprocedural complications (minor or major) were also collected.

**Results:**

Seventy‐five patients received *mild* during the protocol‐defined time period and were included in the study. Only 9 out of 75 patients required lumbar surgical decompression during the 5‐year follow‐up period. Subjects experienced statistically significant pain relief and reduction of opioid medications utilization at 3, 6, and 12 months compared to baseline.

**Conclusion:**

Based on our analysis, the *mild* procedure is durable over 5 years and may allow elderly patients with symptomatic lumbar spinal stenosis to avoid lumbar decompression surgery while providing significant symptomatic relief.

**Key Points**

**What is known**

- While the etiology of lumbar spinal stenosis (LSS) is multifactorial, hypertrophic ligamentum flavum is the primary cause of LSS in the elderly.
- The *mild* procedure allows debulking of the hypertrophic ligamentum flavum without interfering with the integrity of the bony spine, and does not require implants.
- Patients generally present with multiple types of stenosis and numerous spinal comorbidities, and the presence of these cofactors is not an exclusion for treatment with *mild*.
- *mild*, as a minimally invasive lumbar decompression option, represents a safe, effective and cost‐effective alternative with much shorter recovery time.
- *mild* patients experience statistically significant improvement in function and pain through 2‐year follow‐up.

**What this study adds**

- The *mild* procedure significantly decreased the need for surgical decompression at the same treatment level up to 5 years post‐*mild* procedure.
- The incidence of the need for open surgical decompression was a low 2.4% per year after treatment with *mild*.
- Subjects undergoing *mild* procedure had significant pain relief as well as reduced opioids medication use at 3, 6 and 12‐month follow‐up.

## INTRODUCTION

Lumbar spinal stenosis (LSS) is a prevalent spine disorder in the elderly population. It is the most common reason for lumbar spine surgery in patients older than 65 years.^1^ Patients with symptomatic LSS typically experience gradually progressive symptoms of neurogenic claudication, described as lower back and leg pain when walking or standing that is relieved by sitting or bending forward.^2^ These chronic and disabling symptoms lead to limitations of activities of daily life, which impair a patient’s quality of life (QOL).^3^ Diagnosis of LSS depends on the presence of the characteristic clinical picture along with specific radiologic findings namely: the degree of stenosis and the exact spinal level(s), which explain the clinical presentation.

Conservative treatment options are generally offered to patients with LSS first and may include anti‐inflammatory medications, physical therapy, lifestyle modifications, and epidural steroid injections (ESIs). Surgical intervention is recommended when conservative measures fail to improve symptoms. Open surgical techniques include decompression of the lumbar spine, such as laminectomy, partial facetectomy, and discectomy, with and without fusion, which may or may not include hardware instrumentation. However, spine surgery represents a major social and economic burden and poses substantial healthcare risk. It is associated with varying degrees of success and can often include both procedural and anesthesia related complications as well as the potential need for re‐operation.^4^, ^5^

There are a substantial number of patients who have persistent symptoms despite conservative treatments, and likewise are not appropriate candidates for surgery due to high surgical risks or unwillingness to undergo back surgery. The minimally invasive lumbar decompression (*mild* ^®^) procedure (Vertos Medical, Aliso Viejo, CA, USA) represents a safe and effective treatment option for this group of patients.^4^ Although spinal stenosis has many contributing factors, such as intervertebral disc herniation or spondylolisthesis and hypertrophy of facet joints or congenital short pedicles, the majority of elderly patients have hypertrophic ligamentum flavum (HLF) as the primary contributing factor. The *mild* technique offers a minimally invasive, fluoroscopically guided percutaneous debulking of the thickened ligament. It is generally performed under deep sedation, in contrast to spine surgery that may require major incision, instrumentation, and general anesthesia.^5^ The safety and effectiveness of the *mild* procedure have been well established.^2^, ^4^, ^6^, ^7^ In this report, we aim to investigate the long‐term durability of *mild* as measured by the incidence of open lumbar decompression surgery at the same level(s) as the *mild* procedure during 5‐year follow‐up. Our secondary outcomes are change in patient‐reported pain levels as well as opioid medications utilization from baseline to 3, 6, and 12 months post‐*mild* procedure.

## METHODS

### Subject selection

This study was conducted following institutional review board (IRB) approval. Retrospective data was manually collected from the electronic medical records (EMRs) for all patients with LSS with HLF who underwent the *mild* procedure at the Cleveland Clinic Department of Pain Management from January 2010 through December 2015.

### Data collection

Individual EMRs were reviewed to collect demographic data such as age, gender, body mass index (BMI), smoking status, history of substance abuse, alcohol use, psychiatric history, diabetes mellitus, and work/disability status. Alcohol use was classified into the following categories: never used, former drinkers, or current drinkers. In addition, the collected *mild* procedure data included procedure levels and laterality as well as procedural complications (minor or major). Tissue specimens removed during the procedure were sent for histopathology examination to confirm the removal of ligamentum flavum.

### Outcome measures

Our primary outcome was defined as the incidence of open lumbar decompression surgery at the same level(s) as the previously performed *mild* procedure during 5‐year follow‐up. The secondary outcome was change from baseline of the Numeric Rating Scale (NRS) and opioid medications utilization at 3, 6, and 12‐month follow‐up post‐*mild*. NRS is a widely used pain assessment scale ranging from zero to 10 with zero indicating no pain and 10 indicating the worst pain possible. Opioid medications utilization was assessed by the reported Morphine Milligram Equivalent (MME) dose per day.

### Statistical analysis

Descriptive statistics were used for patient demographics and were calculated using the mean ± SD, if normally distributed, and number (%) if not normally distributed.

Multivariate logistic regression was used to analyze the incidence of lumbar spine decompression surgery post‐*mild* procedure, using the covariates of age, gender, BMI, and smoking (ever/never).

The paired Wilcoxon signed‐rank test was used to assess changes between baseline and 3, 6, and 12‐month follow‐up time points for NRS as well as opioid medications utilization measured by MME at the same follow‐up time points. As pain and opioid utilization had imperfect follow‐up at later time points, and were not normally distributed, the paired Wilcoxon signed‐rank test was chosen for its ability to address both of these data issues.

Any result with *p* less than 0.05 was considered statistically significant. All analyses were conducted in Stata version 14.2 (Stata Corp.).

## RESULTS

Seventy‐five patients received the *mild* procedure from 2010 through 2015, and all were included in the analysis. Patient demographic characteristics are summarized in Table 1. This patient population was split fairly equally between women (51%) and men (49%). The mean age was 74.4 years (±9.1 years). The majority of patients were overweight with an average BMI of 29.4 (±5.2). All patients lacked long term symptomatic improvement in response to conservative measures over an average of 6.8 years (±7.6 years) before considering the *mild* procedure. Thirty‐two percent of patients were diabetic. Less than 7% of patients were collecting disability compensation, whereas 93.3% were retired workers. Twelve percent of patients were current alcohol users, 87% never used alcohol, and 1 patient was a former alcohol user. Forty‐nine percent of patients were either current or former smokers. None of these patients were elicit substance abusers, 29% were diagnosed with depression or anxiety, and no patients suffered from post‐traumatic stress disorder (PTSD) or bipolar disorder (Table 1).

**TABLE 1:** Descriptive statistics

Variable	Value
Gender	
Male; *n* (%)	37 (49.3%)
Female; *n* (%)	38 (50.7%)
Age (mean years ± SD)	74.4 ± 9.1
BMI (mean kg/m^2^ ± SD)	29.4 ± 5.2
Duration of pain (mean years ± SD)	6.8 ± 7.6
History of diabetes	
No; *n* (%)	51 (68.0%)
Yes; *n* (%)	24 (32.0%)
Work status	
No; *n* (%)	70 (93.3%)
Yes; *n* (%)	5 (6.7%)
Alcohol status	
Never; *n* (%)	65 (86.7%)
Current	
Moderate; *n* (%)	7 (9.3%)
Heavy; *n* (%)	1 (1.3%)
Not specified; *n* (%)	1 (1.3%)
Former; *n* (%)	1 (1.3%)
Smoking status	
Never; *n* (%)	38 (50.7%)
Current; *n* (%)	5 (6.7%)
Former; *n* (%)	32 (42.7%)
History of substance abuse	
No; *n* (%)	75 (100.0%)
Yes; *n* (%)	0 (0.0%)
Psychiatry history	
No; *n* (%)	53 (70.7%)
Depression; *n* (%)	21 (28.0%)
Anxiety; *n* (%)	1 (1.3%)
PTSD; *n* (%)	0 (0.0%)
Bipolar; *n* (%)	0 (0.0%)

Abbreviations: BMI, body mass index; PTSD, post‐traumatic stress disorder.

One quarter of patients (*n* = 19) underwent the *mild* procedure at two levels and the rest were treated at one level only. The most frequently treated level was L4 to L5 (52.1%; Table 2). There were no major complications recorded. Reported minor complications included post‐procedural soreness and ecchymosis at the surgical site, and one case of allergic dermatitis to the applied surgical tape (Table 3).

**TABLE 2:** *mild* procedure levels

**Total procedure levels**	
L2‐L3; *n* (%)	4 (4.3%)
L3‐L4; *n* (%)	40 (42.6%)
L4‐L5; *n* (%)	49 (52.1%)
L5‐S1; *n* (%)	1 (1.1%)
**Specific levels per patient**	
L2‐L3 only; *n* (%)	2 (2.7%)
L3‐L4 only; *n* (%)	22 (29.3%)
L4‐L5 only; *n* (%)	32 (42.7%)
L5‐S1 only; *n* (%)	0 (0.0%)
L2‐L3 & L3‐L4; *n* (%)	2 (2.7%)
L3‐L4 & L4‐L5; *n* (%)	16 (21.3%)
L4‐L5 & L5‐S1; *n* (%)	1 (1.3%)
**Procedure laterality**	
Unilateral; *n* (%)	10 (13.3%)
Bilateral; *n* (%)	55 (73.3%)
Unilateral and bilateral; *n* (%)	10 (13.3%)

**TABLE 3:** *mild* procedure complications

Complications	*n* (%)
Intra‐operative complications	
No	75 (100.0%)
Yes	0 (0.0%)
Postoperative complications	
None	62 (82.7%)
Minor	13 (17.3%)
Postprocedural pain	11
Ecchymosis	1
Allergic dermatitis	1
Major	0 (0.0%)

The tissue specimens removed during the *mild* procedure and sent for histopathology examination were reported to have dense fibrous connective tissue consistent with ligamentum flavum as well as a few cancellous bone fragments in all 75 cases.

**FIGURE 1:**
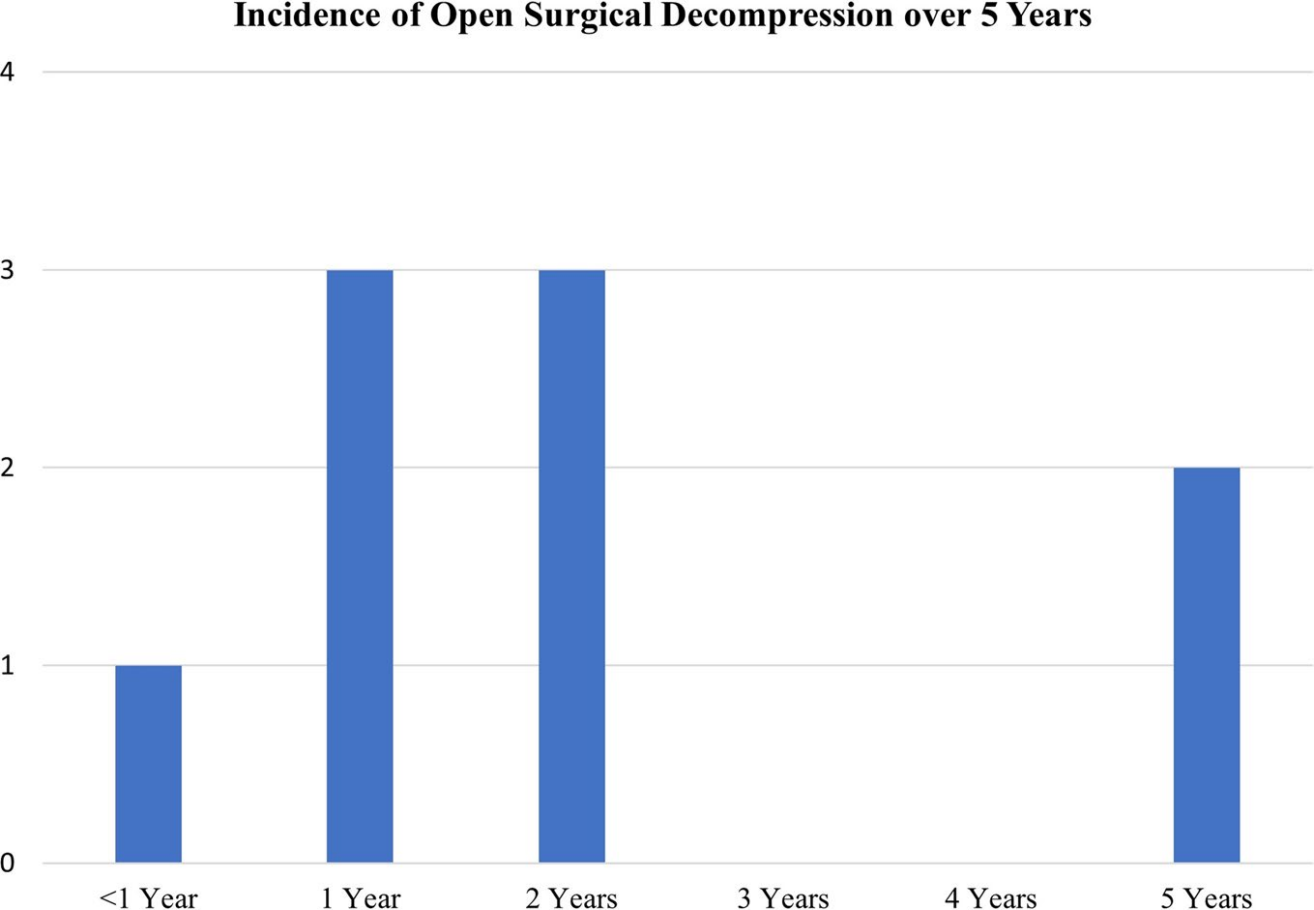
Incidence of open surgical decompression post‐*mild* procedure over 5 years. A total of 9 patients out of 75 (12%) required open surgical decompression within 5 years, representing an annual rate of 2.4%

Three patients were lost to follow‐up, three patients were deceased, and one patient resides outside of the United States. Nine patients out of 75 (12%) required open surgical decompression within the 5‐year follow‐up period (Table 4, Figure 1). This represents an annual incidence of lumbar decompression surgery at the same level as a previously performed *mild* procedure of 2.4%. Logistic regression on the incidence of open surgical decompression, including the covariates of BMI, binary smoking (ever/never), age at the time of the *mild* procedure, and gender, showed no significant predictive ability of patients receiving open surgical lumbar decompression (*p* = 0.2463). Within the logistic model, none of the covariates showed any statistically significant association with the incidence of subsequent open surgery. However, gender was nearly significant, and women had an odds ratio of 0.175 (*p* = 0.053) of having subsequent open surgery when compared with men (Table 5).

**TABLE 4:** Incidence of open surgical decompression during 5 years post‐*mild* procedure

Patient status	*n* (%)
No	59 (78.7%)
Yes	9 (12.0%)
Lost to follow‐up^a^	3 (4.0%)
Deceased^a^	3 (4.0%)
Out of country^a^	1 (1.3%)

Abbreviations: EMR, electronic medical record.

^a^ These subjects had variable follow‐up visits ranging from 6 to 12 months, and, therefore, their pain scores and opioids utilization are incomplete. There is no evidence of back surgery in the medical history of these patients based on EMR inquiries as well as telephone interviews.

**TABLE 5:** Logistic regression on subsequent open surgical decompression

Covariate	Odds ratio	*p* value
BMI	0.960	0.617
Smoking history	1.189	0.817
Age at procedure	0.940	0.185
Gender (female)	0.175	0.053

**FIGURE 2:**
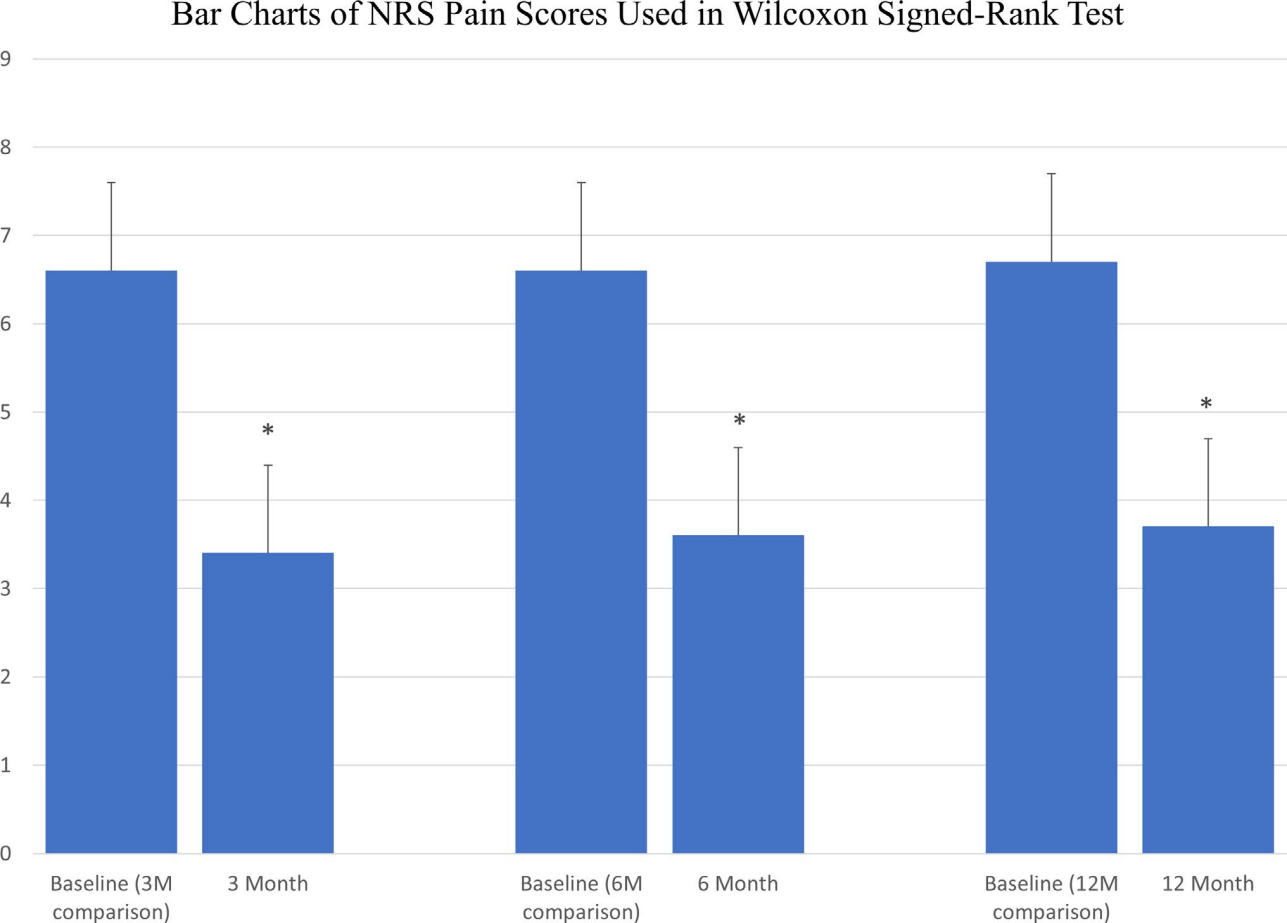
Numeric Rating Scale (NRS) over 12 months. Means and SDs of NRS pain scores reported at baseline, and at 3, 6, and 12 months post‐*mild* procedure demonstrated statistically significant pain relief at all follow‐ups compared to baseline (*p < 0.0001 for each comparison)

**FIGURE 3:**
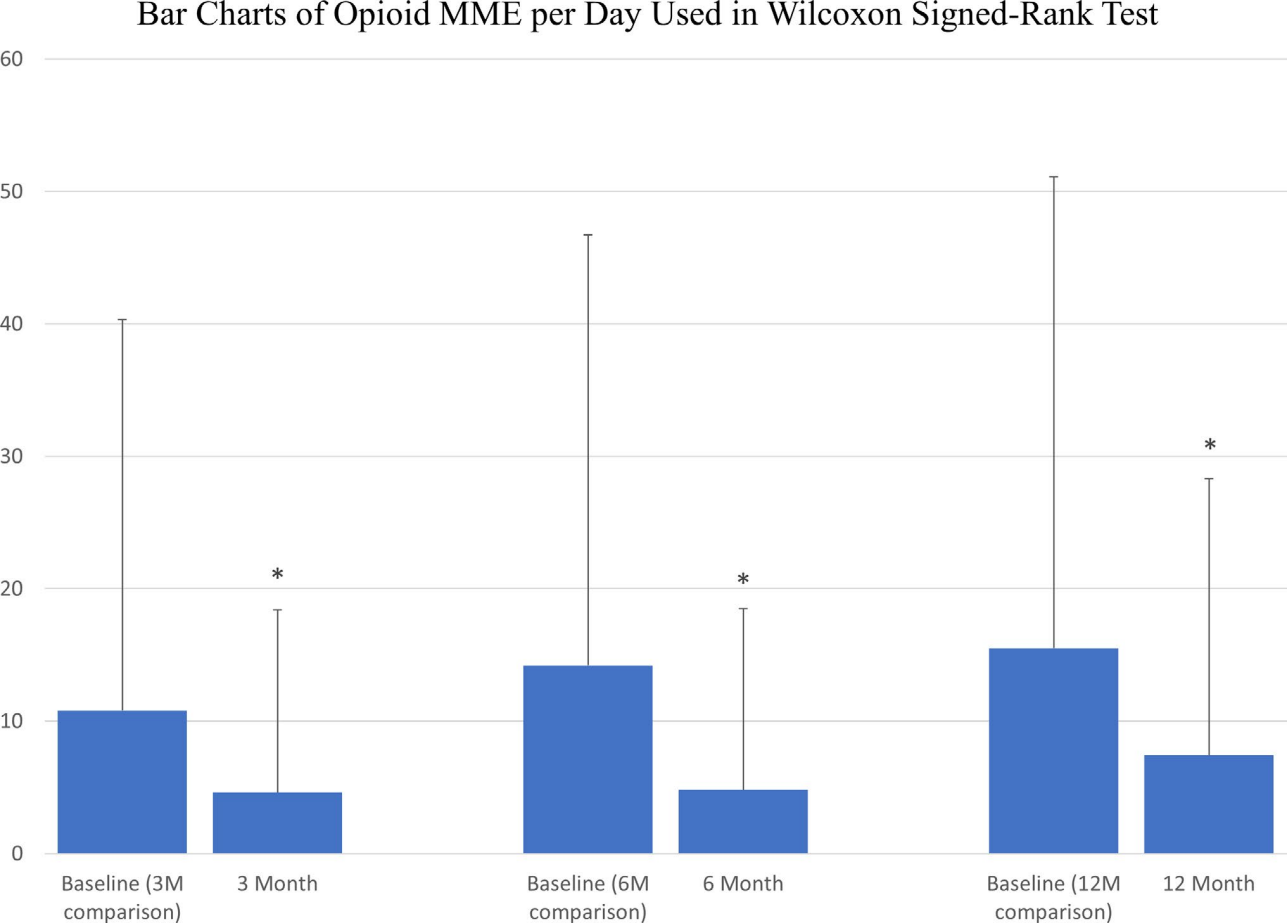
Opioid medications utilization over 12 months post‐*mild* procedure. Means and SDs of opioid MME reported at baseline, and at 3, 6 and 12 months post‐*mild* procedure demonstrated a statistically significant change in opioid medications utilization at all follow‐ups compared to baseline (*p = 0.0048, *p* = 0.0015, and *p* = 0.0067, respectively). The wide SDs are due to the many patients who took no opioids.MME, Morphine Milligram Equivalent

There is a significant difference in the reported NRS pain scores between baseline and all 3 follow‐up time points, including 3, 6, and 12‐months post‐*mild* treatment (*p* < 0.0001 for each time point; Table 6, Figure 2). Although only 24% of subjects (18/75) were treated with opioid medications before the *mild* intervention, there is a statistically significant change in opioid medications utilization between baseline and 3, 6, and 12‐months after *mild* treatment (*p* = 0.0048, *p* = 0.0015, and *p* = 0.0067, respectively; Table 7, Figure 3).

**TABLE 6:** Paired Wilcoxon signed‐rank results: change in NRS pain score at baseline versus follow‐ups

Comparison	*N* in paired comparison	Baseline Mean NRS pain score ± SD^b^	Follow‐up mean NRS pain score ± SD	*p* value for difference	NRS pain reduction of ≥2 *N* (%)	Cohen’s *d* ^c^
Baseline^a^ vs. 3 months	65	6.6 ± 2.2	3.4 ± 2.7	<0.0001	48 (73.8%)	1.08
Baseline^a^ vs. 6 months	59	6.6 ± 2.2	3.6 ± 2.9	<0.0001	41 (69.5%)	0.89
Baseline^a^ vs. 12 months	64	6.7 ± 2.2	3.7 ± 2.8	<0.0001	45 (70.3%)	0.94

Abbreviation: NRS, Numeric Rating Scale.

^a^ The full baseline dataset is 75 patients, with a mean (SD) of 6.6 (2.2).

^b^ Baseline descriptive statistics are for the baseline subset in the paired comparison.

^c^ A Cohen’s *d* value of 0.2 indicates a small effect size, a value of 0.5 indicates a medium effect size, and a value of 0.8 indicates a large effect size.

**TABLE 7:** Paired Wilcoxon signed‐rank results: change in opioid MME per day at baseline versus follow‐ups

Comparison	*N* in paired comparison	Baseline mean opioid MME per day ± SD^b^	Follow‐up mean opioid MME per day ± SD^b^	*p* value for difference	*N* with opioid reduction (mean MME reduction)
Baseline^a^ vs. 3 months	49	10.8 ± 29.5	4.6 ± 13.8	0.0048	13 (27.9)
Baseline^a^ vs. 6 months	40	14.2 ± 32.5	4.8 ± 13.7	0.0015	14 (28.0)
Baseline^a^ vs. 12 months	32	15.5 ± 35.6	7.4 ± 20.9	0.0067	12 (24.2)

Abbreviation: MME, Morphine Milligram Equivalent.

^a^ The full baseline dataset is 75 patients, with a mean (SD) of 7.6 (24.6).

^b^ Baseline descriptive statistics are for the baseline subset in the paired comparison.

When surgical decompression is compared to *mild* treatment levels, only three of nine patients had the surgical decompression exactly at the *mild* procedure levels, whereas the rest (6 of 9), received open surgical decompression at more levels than the *mild* procedure (Table 8). Postoperative follow‐up visits for open surgical decompression patients showed that only two patients (22%), reported improvement of the neurogenic claudication. Three patients (33%) reported no improvement after surgery and one (11%) experienced worsening of pain. Three patients (33%) did not have follow‐up visits after open decompressive surgery (Table 9). One of the nine surgical patients required repeat lumbar spinal surgery (Table 8).

**TABLE 8:** *mild* procedure levels versus open decompression surgery levels for patients who underwent subsequent surgical decompression

Re‐operation patients	*mild* procedure	Subsequent open surgical decompression	
	Levels treated	Levels treated	Type of surgical intervention
1	L3‐L4, L4‐L5	L3‐L5	Laminectomy and fusion
2	L3‐L4, L4‐L5	L1‐L5	Laminectomy and fusion
3	L3‐L4	L3‐L5	Laminectomy and fusion
4	L3‐L4	L3‐L5	Laminectomy
5	L4‐L5	L3‐L5	Laminectomy
6	L4‐L5	L4‐L5	Laminectomy
7	L4‐L5	L2‐S1	Laminectomy and fusion
8	L4‐L5	L4‐L5	Laminectomy
9	L4‐L5	First surgery: L3‐L5 Second surgery: L2‐L4	First surgery: laminectomy and fusion Second surgery: fusion

**TABLE 9:** Pain relief after open surgical decompression

Change in pain level	*n* (%)	Follow‐up
Improvement	2 (22%)	2 and 5 months
Worsening of pain	1 (11%)	1.5 months
No pain relief	3 (33%)	2, 3, and 6 months
Lost to follow‐up	3 (33%)	N/A

Abbreviation: N/A, not applicable.

## DISCUSSION

Both prevalence and severity of acquired spinal stenosis have been reported to increase with age. Specifically, the prevalence of acquired LSS was found to be 47.2% for relative LSS and 19.4% for absolute LSS in the 60‐ to 69‐year‐old age group. Further, LSS is associated with a three‐fold higher risk of experiencing low back pain.^8^ Although the etiology of LSS is multifactorial, thickened ligamentum flavum remains the main cause of LSS in the elderly and accounts for up to 85% of spinal canal narrowing.^9^ The main presenting symptom is neurogenic claudication causing functional disability and impaired QOL. In fact, a literature review by Otani et al. demonstrates that LSS has more negative impact on QOL than cardiovascular and cerebrovascular diseases.^3^ Diagnosis and successful treatment result in significantly enhanced functionality and markedly improved QOL.^2^, ^4^

Current treatment options range from conservative management, such as anti‐inflammatory medications and physical therapy with or without ESIs, to invasive decompressive spine surgery.^2^, ^4^ Conservative measures generally fail to provide long‐term relief for the patients with moderate‐to‐severe LSS and, thus surgery is required most of the time. The goal of surgical treatment for symptomatic LSS is to alleviate neurogenic claudication symptoms by decompressing the lumbar spine and relieving compression of the cauda equina. Surgical modalities for open decompression include laminectomy, foraminotomy, or discectomy with possible additional fusion with or without instrumentation to preserve the stability of the lumbar spine. LSS is, in fact, the most common reason for lumbar spine surgery in patients over 65 years of age, with an economic burden of $1.65 billion per year.^10^, ^11^

The incidence of complex lumbar fusion surgery has been steadily increasing over the past 2 decades, from 7.5 per 100,000 in the year 2000 to 17.8 per 100,000 in 2009.^11^ Although open surgical decompression is associated with good outcomes in two thirds of patients, potential complications of open surgery include local tissue trauma, prolonged postoperative pain, and numerous neurological sequelae, such as sensory deficits, bowel and bladder dysfunction, and the need for additional subsequent surgeries.^12^ These are major concerns when treating elderly patients who often present with multiple comorbidities.^6^ The two most commonly reported complications of decompressive surgery are bleeding requiring blood transfusion and dural tears. Weinstein et al. estimated the incidence to be 14.3% and 9.4%, respectively.^13^ When compared with other lumbar spine interventions, including surgical decompression, spinal fusion, and interspinous spacers, re‐operation and spinal fracture rates are lower, and safety is higher for *mild*.^7^ *mild*, as a minimally invasive lumbar decompression option, represents a safe, effective, and cost‐effective alternative with a much shorter recovery time.^5^, ^7^ The *mild* procedure allows debulking of the hypertrophic ligamentum flavum without interfering with the integrity of the bony spine, and does not require implants.^4^, ^5^ This supports the robust safety profile of the *mild* procedure as compared with open decompression spine surgery as well as interspinous process decompression.^13^, ^14^

Patients who underwent the *mild* procedure and were included in this cohort suffered from neurogenic claudication and central spinal stenosis due to ligamentum flavum hypertrophy. These patients were not experiencing radicular pain defined as pain in the distribution of lumbo‐sacral roots, had no motor weakness, no bowel or bladder dysfunction, and did not exhibit spondylolisthesis greater than grade 1 or instability on dynamic spine x‐rays. Patients generally present with multiple types of stenosis and numerous spinal comorbidities, and the presence of these cofactors is not an exclusion for treatment with *mild*. Specifically, for patients presenting with lateral recess stenosis, debulking the ligamentum flavum from medial to lateral at the end of the procedure may contribute to improved leg pain.

A prospective study revealed statistically significant functional improvement and decreased disability after the *mild* procedure. In this study, patients did not experience any significant procedure related adverse events.^2^ In a large prospective randomized study, patients who underwent the *mild* procedure experienced significant improvement in all outcome measures including Oswestry Disability Index (ODI), Numeric Pain Rating Scale (NPRS), and Zurich Claudication Questionnaire (ZCQ) at 1‐year and 2‐year follow‐up, and the safety of *mild* was shown to be similar to ESIs.^15^ Eight of 143 patients (5.6%) who underwent the *mild* procedure required spinal surgery during the 2‐year follow‐up period.^7^ Similarly, our report demonstrates a low percentage of patients resorting to open surgical decompression options after undergoing the *mild* procedure; 9.3% at 2 years and 12.0% at 5‐year follow‐up (Figure 1). This represents an annual incidence rate of open lumbar surgical decompression of only 2.4%.

The incidence of lumbar surgery after the *mild* procedure showed no significant association with any of the demographic covariates we explored, including age, gender, BMI, and smoking status. However, gender almost reached our significance threshold (*p* = 0.053), and we expect that with a larger sample size, it would reach significance. If the observed odds ratio in our model were maintained within a larger sample size, the results of our logistic regression suggest that women would likely have about 82.5% decreased odds of follow‐up lumbar surgery within 5 years versus men, when holding BMI, smoking status, and age constant (Table 5).

Open surgical treatment for LSS may eventually lead to repeat surgical interventions. Multiple studies looked at the re‐operation rate after spine surgery for LSS. In a nationwide cohort study, the re‐operation rate after decompression with fusion for LSS without concurrent spondylolisthesis was 20% at 5 years. At 3 months, the cumulative re‐operation rate was 4.7% and 7.2%, 9.4%, 11.2%, 12.5%, and 14.2% at 1, 2, 3, 4, and 5 years, respectively. The adjusted re‐operation rate was similar for decompression and fusion surgeries (*p* = 0.82). The calculated re‐operation rate at 10 years was estimated to be 22.9%.^16^ Lad et al. retrospectively studied patients with spinal stenosis without spondylolisthesis who underwent spinal surgery between 2002 and 2009. The long‐term re‐operation rate for decompression alone was not significantly different from combined fusion and decompression at 5 years (17.3% vs. 16.0%, respectively, *p* = 0.44). Similarly, there was no difference in the total cost for the surgery, hospitalization, emergency department, and medications. The fusion with instrumentation re‐operation rate appeared to be higher than without instrumentation; however, the difference was not statistically significant (17.4% vs. 12.2%, respectively; *p* = 0.11). Surprisingly, there was no difference in the total costs whether instrumentation was used or not ($107,056 and $100,471).^17^ A systematic review by Yavin et al. demonstrated that compared to decompression alone, fusion was associated with more complications and increased risk of re‐operation. A meta‐analysis was conducted to assess the available evidence in regard to lumbar decompression alone, lumbar fusion, and nonsurgical care. The risk of re‐operation was significantly higher for spinal stenosis (relative risk [RR] = 1.17, 95% confidence interval [CI] = 1.06–1.28) than for spondylolisthesis (RR = 0.75, 95% CI = 0.68–0.83).^11^ A study of 5‐year durability of interspinous process decompression reported a 25% re‐operation rate at 5‐year follow‐up. This high incidence of re‐operations for interspinous spacer patients was also evident at 12‐month follow‐up when 14.2% of patients who received a spacer had already undergone a re‐operation.^14^ It is worth noting that interspinous process decompression requires the use of an implanted distraction device that is placed between the patient’s vertebral spinous processes to restrict extension.

The present study demonstrated a lower incidence of lumbar surgery (2.4% per year) in a span of 5‐year follow‐up. Therefore, one may conclude that the *mild* procedure saved 88% of patients with symptomatic LSS from open lumbar decompression surgery for at least 5 years. At the same time, the need for open lumbar decompression surgery after *mild* occurs at a much lower rate than the rate of re‐operation after lumbar spine surgery. Of note also, seven of nine subjects received open surgical decompression at more levels than the *mild* procedure (Table 8). Yet, only two patients (22%), reported improvement of the neurogenic claudication. Three patients (33%) reported no improvement after surgery and one (11%) experienced worsening of pain. That underscores the durability and effectiveness of the *mild* procedure in relieving the neurogenic claudication in the appropriate patients. Further, because the *mild* procedure demonstrated durability up to 5 years, it might also be speculated with caution, that appropriate patients should be encouraged to undergo the *mild* procedure as early as needed, rather than waiting until these patients are at an advanced age.

An analysis by Udeh et al. shows a cost of $43,760 per quality‐adjusted life years (QALY) for the *mild* procedure at 2 years versus $125,985 per QALY for laminectomy.^5^ In another study by Tosteson et al., lumbar surgical decompression significantly improved the QOL of patients with spinal stenosis at a cost of $77,600 (CI = $49,600 to $120,000) per QALY gained in comparison with nonsurgical management. As for fusion in degenerative spondylolisthesis, the adjusted cost was $115,600 (CI = $90,800 to $144,900) per QALY gained.^18^ Thus, *mild* seems to be a more cost‐effective option for LSS compared to open lumbar decompression surgery and other surgical treatment options.

The ability to avoid lumbar spine surgery in our cohort was justified with meaningful analgesia. The NRS pain score showed significant improvement between baseline and 3, 6, and 12‐month follow‐ups. Further justification in the form of decreased opioid medications use was also seen between baseline and 3, 6, and 12‐month follow‐ups. Our anecdotal clinical observations were that loss to follow‐up was nonrandom, and especially after 12 months, those who had the best *mild* outcomes were the least likely to return for follow‐up visits. This can be seen in the fact that the 12‐month data included 85% of treated patients, and the rate of follow‐up visits after the first year decreased significantly to about 33% at 24 months. With any loss of greater than or equal to 20% outcome data at any follow‐up time point, there is significant potential for bias. For that reason, we decided to report the changes in NRS and opioid medications only up to 12 months.

Our study bears all limitations of retrospective data analysis. However, every effort was made to ensure the accuracy of data. Telephone calls were made to confirm data if needed. Possible other confounding factors affecting the incidence of subsequent open surgery, reported pain scores, and opioid consumption may not have been measured. Missing follow‐up data for a few patients may still pose a limitation for our analysis.

## CONCLUSION

In this study, the *mild* procedure significantly decreased the need for surgical decompression at the same treatment level at 2 and 5 years post‐*mild* procedure. This objective measure provides a real‐world view of the success of *mild* related to long‐term patient outcomes, as well as lower utilization of relatively higher‐cost back surgery. Significant pain relief and functional improvement after *mild* has been supported in the literature. The durability of *mild* over 5 years may allow elderly patients with symptomatic lumbar spinal stenosis to avoid lumbar decompression surgery while providing significant symptomatic relief. This highlights the potential role of *mild* to significantly impact patients’ QOL while avoiding a major health and economic burden.

## CONFLICT OF INTEREST

N.M. reports functioning as an independent medical monitor for the MOTION multi‐center randomized controlled study sponsored by Vertos Medical. S.C. reports functioning as Cleveland Clinic Principal Investigator for the MOTION multicenter randomized controlled study sponsored by Vertos Medical.
